# Improving the accuracy of cotton seedling emergence rate estimation by fusing UAV-based multispectral vegetation indices

**DOI:** 10.3389/fpls.2024.1333089

**Published:** 2024-03-27

**Authors:** Tiansheng Li, Haijiang Wang, Jing Cui, Weiju Wang, Wenruiyu Li, Menghao Jiang, Xiaoyan Shi, Jianghui Song, Jingang Wang, Xin Lv, Lifu Zhang

**Affiliations:** ^1^ College of Agriculture, Shihezi University, Shihezi, China; ^2^ Aerospace Information Research Institute, Chinese Academy of Sciences, Beijing, China

**Keywords:** machine vision, vegetation index, machine learning, Otsu, multispectral image

## Abstract

Timely and accurate estimation of cotton seedling emergence rate is of great significance to cotton production. This study explored the feasibility of drone-based remote sensing in monitoring cotton seedling emergence. The visible and multispectral images of cotton seedlings with 2 - 4 leaves in 30 plots were synchronously obtained by drones. The acquired images included cotton seedlings, bare soil, mulching films, and PE drip tapes. After constructing 17 visible VIs and 14 multispectral VIs, three strategies were used to separate cotton seedlings from the images: (1) Otsu’s thresholding was performed on each vegetation index (VI); (2) Key VIs were extracted based on results of (1), and the Otsu-intersection method and three machine learning methods were used to classify cotton seedlings, bare soil, mulching films, and PE drip tapes in the images; (3) Machine learning models were constructed using all VIs and validated. Finally, the models constructed based on two modeling strategies [Otsu-intersection (OI) and machine learning (Support Vector Machine (SVM), Random Forest (RF), and K-nearest neighbor (KNN)] showed a higher accuracy. Therefore, these models were selected to estimate cotton seedling emergence rate, and the estimates were compared with the manually measured emergence rate. The results showed that multispectral VIs, especially NDVI, RVI, SAVI, EVI2, OSAVI, and MCARI, had higher crop seedling extraction accuracy than visible VIs. After fusing all VIs or key VIs extracted based on Otsu’s thresholding, the binary image purity was greatly improved. Among the fusion methods, the Key VIs-OI and All VIs-KNN methods yielded less noises and small errors, with a RMSE (root mean squared error) as low as 2.69% and a MAE (mean absolute error) as low as 2.15%. Therefore, fusing multiple VIs can increase crop image segmentation accuracy. This study provides a new method for rapidly monitoring crop seedling emergence rate in the field, which is of great significance for the development of modern agriculture.

## 1 Introduction

Natural disasters such as sandstorms are prone to occur during cotton emergence stage in China’s main cotton growing areas, causing germination failure and even death (Knowles and Knowles, 2016; Chen et al., 2018; He et al., 2020). Xinjiang is the largest cotton growing base in China (Wang et al., 2018), with cotton yield accounting for 89.50% of China’s total in 2021. Timely and accurate estimation of cotton seedling emergence rate is of great significance for post-disaster agricultural management and yield assessment. Traditionally, manual counting has been used to monitor crop emergence (Jin et al., 2017), which is time-consuming, labor-intensive, making it unsuitable for large-scale monitoring (Wiles and Schweizer, 1999). Therefore, there is an urgent need for fast, convenient and accurate monitoring methods.

Remote sensing technology has been widely applied in the monitoring of crop growth, insect pests and diseases, and yield estimation (Duan et al., 2017). Thorp et al. (2006) and Liu et al. (2017a) used satellites and ground vehicles equipped with optical sensors to monitor crop seedling emergence rate in farmlands, and found that the accuracy of satellite monitoring was not high due to the influences of spatial resolution, cloud cover, and revisit period (Varela et al., 2018). Besides, due to the influences of ground flatness, soil humidity, and driving speed (Banerjee et al., 2021), the accuracy of ground vehicle-based monitoring was also unsatisfactory. Drones (Zhou et al., 2021), a flexible and portable platform, are less affected by aerosols and ground conditions, and can address the shortcomings of satellite and ground vehicle platforms in agricultural monitoring (Kasampalis et al., 2018). The use of drones has achieved accurate monitoring of crop nutrition status (Liu et al., 2017a), growth parameters (Zhu et al., 2019), diseases (Ballester et al., 2017), and yield estimation (Feng et al., 2020b).

At present, template matching is widely used in the monitoring of crop seedling emergence based on drone-based remote sensing (Banerjee et al., 2021). Specifically, VI is constructed to highlight crop information. Then, crop seedlings are separated from images using Otsu thresholding algorithm (Varela et al., 2018). After that, crop morphological characteristics (such as axis length, roundness, and area) are extracted, to construct a standard template of crop seedling morphology (García-Martínez et al., 2020). Finally, crop seedlings are identified by comparing all objects in the image with the standard template (Li et al., 2019). Li et al. (2019) used six morphological features as inputs of a random forest model to estimate potato seedling emergence rate, and found that the correlation coefficient was as high as 0.96. Zhao et al. (2018) extracted 15 features of rapeseed, and found that these features had a strong correlation with the measured number of seedlings, with a coefficient of determination (R^2^) of 0.867 and a MAE of 5.11%. However, with the growth of crops, the morphological characteristics of crop seedlings continue to change, leading to a decrease in the timeliness of standard templates (García-Martínez et al., 2020). This ultimately affects the segmentation accuracy.

Visible sensors have been widely used in monitoring crop seedling emergence because of cheapness (Li et al., 2019). However, visible sensors only has three channels [Red, Green, Blue (RGB)], causing difficulty in distinguishing surface features with similar colors (Banerjee et al., 2021). To extract the information of RGB images, some researchers have constructed visible VIs based on the color of crop leaves, with ExG (Excess Green Index) widely praised (Liu et al., 2018). Some scholars have converted RGB color spaces to other color spaces, such as HSV (Hue, Saturation, Value) and CIELAB components (Dai et al., 2020), to highlight the features of crop seedlings in images. These methods can increase the estimation accuracy of emergence rate based on visible images, but they are limited by the number of channels in images and the wide band of visible sensors. However, multispectral sensors have a narrow band and can extract more information (Li et al., 2019). Besides, the VIs constructed based on multispectral sensor data are less affected by light changes. Thus, multispectral sensors present a higher accuracy in crop seedling emergence monitoring (Feng et al., 2020a).

Separating crops from images based on differences in spectral reflectance of different image features is the key to improving seedling emergence monitoring accuracy. In the segmentation of features in satellite multispectral images, researchers usually use machine learning to perform pixel-level segmentation based on differences in reflectance. For example, Xun et al. (2021) constructed a model based on the fusion of spectral data to monitor the main cotton growing areas of China, and the R^2^ of the estimates and measured values was 0.83. Zhang et al. (2019) constructed a random forest model to monitor wheat growing areas in northern and central Anhui Province, showing an accuracy of 93% ~ 97% for northern Anhui and 80% for central Anhui. Different from the segmentation of satellite multispectral images, researchers tend to use machine vision technology to segment drone-based remote sensing images to extract crop information (Meyer and Neto, 2008). For example, Varela et al. (2018) used Otsu thresholding algorithm to extract maize information from ExG images. Liu et al. (2017b) converted RGB images of wheat farmland into CIELAB color space and performed threshold segmentation on vector a to extract wheat seedling information.

At present, there are few researches on the use of drones to monitor crop seedling emergence. While some studies have applied machine vision technology to crop canopy image segmentation and classification, there are few studies using multispectral sensors for crop canopy image segmentation. Therefore, there is a huge space to improve crop image segmentation. In this study, remote sensing images of cotton fields in Xinjiang, China were acquired during seedling stage (2 - 4 leaves) to construct 31 VIs. Then, the effects of single VI and multiple VI fusions on cotton seedling image segmentation accuracy by Otsu thresholding algorithm were compared. Finally, the cotton seedling emergence rates in the study area were visualized. This study will provide a new technical tool for monitoring crop seedling emergence.

## 2 Materials and methods

### 2.1 Study site

**Figure 1:**
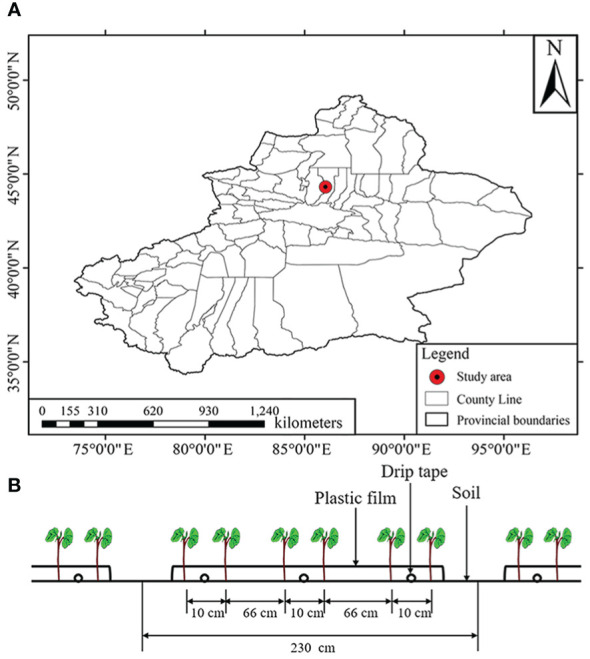
Overview of the study site. **(A)** Location of the study site; **(B)** Cotton planting pattern in Xinjiang.

The experiment was conducted at the Erlian Experimental Site of Shihezi University, Xinjiang, China (44°18’ N, 86°03’ E, a.s.l. 440 m) (**Figure 1A**) in 2021. The region has a temperate continental climate, with large evaporation and little rainfall. The average annual precipitation was 180 - 270 mm and the average annual temperature was 7°C in 2021. On May 1, 2021, a dust storm hit the area, reducing the visibility to a minimum of 590 m. During the dust storm, the seed holes were highly susceptible to wind erosion, resulting in a decrease in seedling emergence. The widely planted cotton (*Gossypium* spp) cultivar in Shihezi, Xinluzao No. 64, was used in this experiment. The planting pattern designed for machine harvest was adopted (**Figure 1B**), that is, six rows were irrigated with three drip tapes under the mulching of one film. The row spacing configuration was 66 + 10 cm, the plant spacing was 10 cm, and the plant density was 260,000 plants per hectare. Drip irrigation was employed. The PE drip tape spacing was 76 cm, the emitter spacing was 30 cm, and the drip flow rate was 1.8 L/h. Cotton seeds were sown and drip-irrigated (300 m^2^/ha) on April 26, 2021.

### 2.2 Data acquisition

#### 2.2.1 Measurement of cotton seedling emergence rate

**Figure 2:**
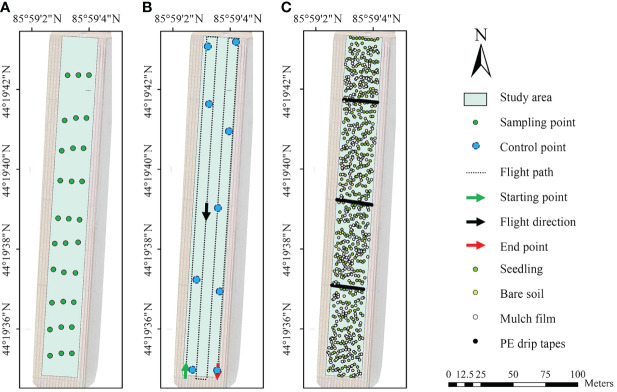
Location of sampling points for measuring emergence rate **(A)**, flight route **(B)**, and **(C)**. Location of the pixels of cotton seedlings, bare soils, mulch films, and PE drip tapes in the acquired image.

According to the cotton planting pattern (Feng et al., 2017) and the suggestion of the image acquisition time of Zhao et al. (2018), the emergence rate were acquired at the seedling stage (plants had 2 - 4 leaves) (May 9, 2021). At this stage, the canopy diameter of each cotton seedling was 3 - 6 cm, while the plant spacing was 10 cm. Therefore, there were almost no overlapping leaves. Thirty sampling plots (2.3 × 2.3 m) were selected along a S-shaped line (**Figure 2A**), then the coordinate of the center of each plot were recorded. The number of seedlings in each plot was manually counted, and the emergence rate was calculated based on the seeding rate.

#### 2.2.2 Drone-based image acquisition

After manually counting the seedlings in the field, images of the entire cotton field were acquired using the Phantom 4 Mutispectral drone (DJI, Shenzhen, China) from 12:00 to 14:00. The drone was equipped with one visible camera and five multispectral cameras, which could acquire RGB and multispectral images at the same time. Therefore, the images obtained by the six cameras had only a small position offset at the hardware level. The drone calculated the position offset of each camera from the NIR camera, and this offset was included into the metadata of the images and used in the GPS correction. Therefore, the GPS information for the six cameras were not consistent. The CMOS size of a single camera was 1/2.9 inch, and the focal length was 5.74 mm. The bands of the acquired images are shown in **Table 1**.

**Table 1:** Spectral bands acquired by the drone-based sensors.

Sensor	Band range
Visible	400 nm - 700 nm
Blue	450 nm ± 16 nm
Green	560 nm ± 16 nm
Red	650 nm ± 16 nm
Red-edge	730 nm ± 16 nm
Nir-red	840 nm ± 26 nm

The DJI GS Pro software (DJI, Shenzhen, China) was used to design flight path (**Figure 2B**). Lenses were vertically downward during flight to take images at equal intervals. The flight altitude was 30 meters, The ground sampling distance (GSD) of the images was 1.607 cm pixel^-1^ which theoretically sufficient to obtain clear UAV images, and the longitudinal overlap and side overlap were 75%. The shutter time was 1/20000 s for the visible camera and AUTO for multispectral cameras. Reference board (MAPIR, USA) was placed horizontally in an unconcluded position, so that it appeared in the images taken by the drone.

A total of 1614 images (269 images for each camera) were obtained, and the size of each image was 1600 × 1300 pixels. The format of visible images was JPG, and that of multispectral images was TIFF.

### 2.3 Data processing

**Figure 3:**
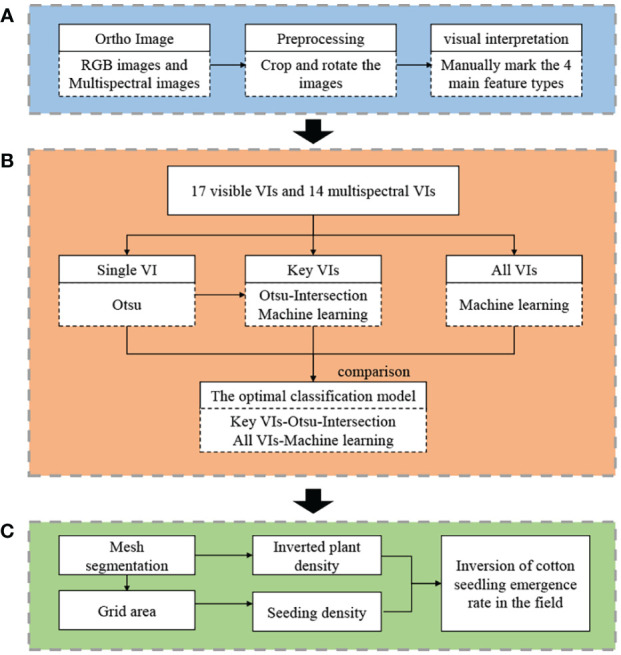
Flow chart of drone-based monitoring of cotton seedling emergence rate. **(A)** Image preprocessing and visual interpretation; **(B)** Extraction of cotton seedlings from images; **(C)** Inversion of seedling emergence rate.

The acquired images were processed with the following procedures (**Figure 3**): (A) Image preprocessing and visual interpretation. The acquired orthophoto images were rotated, cropped, and corrected. By visual interpretation, the features in the images were classified into cotton seedlings, bare soils, mulch films, and PE drip tapes, and the corresponding pixels were randomly selected. Besides, the coordinates were also extracted. (B) Extraction of cotton seedlings from the images. According to the results of previous researches, 17 visible VIs and 14 multispectral VIs were constructed based on 1 visible image and 5 multispectral images, and three strategies were used to extract: (1) Otsu’s thresholding was conducted on each VI; (2) Key VIs were selected based on the results of (1), and the intersection between two binary images and three machine learning methods were used to classify the features in the images; (3) Machine learning classification models were constructed using all VIs and validated. (C) Inversion of cotton seedling emergence rate. The images were morphologically filtered and divided into consistent grids. The number of cotton seedlings in each grid was counted, and the inversion accuracy was verified according to the manually measured seedling emergence rate.

### 2.4 Data preprocessing

The acquired images were stitched together using Pix4D mapper software (Pix4D, Lausanne, Switzerland), When stitching the images, each band has at least 6 photos to be marked to a control point, to ensure the consistency of images in the GPS coordinates. Then, the georeferencing tool in the ENVI Classic 5.3 (Harris Geospatial; Broomfield, Colorado, USA) software was used for georeferencing of images, using the NIR image as the reference image, all above can reduce the pixel-scale error of images. Finally, the radiometric correction, combination, rotation, cropping, and other operations were carried out.

The main features in the images were cotton seedlings, bare soils, mulch films, and PE drip tapes. Through visual interpretation, the location of pixels in different features was manually randomly labeled with the region of interest (ROI) tool, to make the sampling points evenly distributed in the study area (**Figure 2C**). After exporting the data (labeled coordinates, reflectance, and DN value), the images and coordinates were imported into MATLAB and checked after re-export, to ensure that the data exported by MATLAB were the same as those exported by ENVI. A total of 302, 224, 237, and 200 pixels were labeled for cotton seedlings, bare soils, mulch films, and PE drip tapes, respectively.

### 2.5 Extraction of cotton seedlings from images

#### 2.5.1 Construction of VIs

According to the sampling bands and band ranges of the sensors, combined with the results of previous researches, 17 visible VIs and 14 multispectral VIs related to plant leaves (**Table 2**) were selected.

**Table 2:** Selected vegetation indices for this study.

Index	Expression	References
Visible VIs		
r	$r=\;\frac{R}{R+G+B}$	Finlayson et al. (1998)
g	$g=\;\frac{G}{R+G+B}$	Finlayson et al. (1998)
b	$b=\;\frac{B}{R+G+B}$	Finlayson et al. (1998)
NGRDI	$NGRDI=\frac{g-r}{g+r}$	Tucker (1979)
GLI	$GLI=\frac{2\times g-r-b}{2\times g+r+b}$	Louhaichi et al. (2001)
ExR	ExR=1.4×r−g	Meyer et al. (1999)
ExG	ExG=2×g−r−b	Woebbecke et al. (1995)
ExB	ExB=1.4×b−g	Mao et al. (2003)
ExGR	ExGR=ExG−ExR	Meyer and Neto (2008)
CIVE	CIVE=0.411×R−0.811×G+0.385×B+18.78745	Kataoka et al. (2003)
VEG	$VEG=\frac{G}{R^{\alpha }\times B^{\left(1-\alpha \right)}}\left(\alpha =0.667\right)$	Hague et al. (2006)
MExG	MExG=1.262×G−0.884×R−0.311×B	Tang et al. (2003)
IKAW	$IKAW=\frac{R-B}{R+B}$	Kawashima (1998)
TGI	TGI=0.5×(0.19×(R−G)−0.12×(R−B))	Hunt et al. (2011)
COM1	COM1=ExG+CIVE+ExGR+VEG	Guijarro et al. (2011)
COM2	COM2=0.36×ExG+0.47×CIVE+0.17×VEG	Guerrero et al. (2012)
Muti-spectral Vis		
NDVI	$NDVI\;=\;\frac{\rho_{NIR}-\rho_{Red}}{\rho_{NIR}+\rho_{Red}}$	Rouse et al. (1974)
GNDVI	$GNDVI\;=\;\frac{\rho_{NIR}-\rho_{Green}}{\rho_{NIR}+\rho_{Green}}$	Gitelson et al. (1996)
RVI	$RVI\;=\;\frac{\rho_{NIR}}{\rho_{Red}}$	Jordan (1969)
DVI	$DVI\;=\rho_{NIR}-\rho_{Red}$	Jordan (1969)
RDVI	$RDVI\;=\;\frac{\rho_{NIR}-\rho_{Red}}{\sqrt{\rho_{NIR}+\rho_{Red}}}$	Roujean and Breon (1995)
SAVI	$SAVI\;=\;\frac{\left(1+L\right)\times (\rho_{NIR}-\rho_{Red})}{\rho_{NIR}+\rho_{Red}+L},$ (L = 0.5)	Huete (1988)
CI	$CI\;=\;\frac{\rho_{NIR}}{\rho_{Green}}-1$	Gitelson et al. (2003)
NLI	$NLI\;=\;\frac{\rho_{NIR}^{\;2}-\rho_{Red}}{\rho_{NIR}^{\;2}+\rho_{Red}}$	Goel and Qin (1994)
MNLI	$MNLI\;=\;\frac{\left(1+L\right)\times (\rho_{NIR}^{\;2}-\rho_{Red})}{\rho_{NIR}^{\;2}+\rho_{Red}+L}$ (L= 0.5)	Gong et al. (2003)
TVI	$TVI\;=60\times (\rho_{NIR}-\rho_{Green})-100\times (\rho_{Red}-\rho_{Green})$	Broge and Leblanc (2001)
EVI2	$EVI2\;=\;\frac{2.5\times \left(\rho_{NIR}-\rho_{Red}\right)}{\rho_{NIR}+2.4\times \rho_{Red}+1}$	Jiang et al. (2008)
MSAVI2	$MSAVI2=\frac{2\times \;\rho_{NIR}+1-\sqrt{{\left(2\times \rho_{NIR}+1\right)}^{2}-8\times \left(\rho_{NIR}-\rho_{Red}\right)}}{2}$	Qi et al. (1994)
OSAVI	$OSAVI\;=\;\frac{\rho_{NIR}-\rho_{Red}}{\rho_{NIR}+\rho_{Red}+0.16}$	Rondeaux et al. (1996)
MCARI	$MCARI2=\frac{1.5\times \left(2.5\times (\rho_{NIR}-\rho_{Red})-1.3\times (\rho_{NIR}-\rho_{Green})\right)}{\sqrt{{\left(2\times \rho_{NIR}+1\right)}^{2}-6\times (\rho_{NIR}-5\times \rho_{Red})-0.5}}$	Haboudane et al. (2004)

R, G, B represent the values of the images acquired by the red, green, and blue channels by visible camera, respectively, and ${\text{ρ}}_{\text{Blue}}$ , ${\text{ρ}}_{\text{Green}}$ , ${\text{ρ}}_{\text{Red}}$ , ${\text{ρ}}_{\text{Red}-\text{edge}}$ , and ${\text{ρ}}_{\text{Nir}}$ represent the reflectance of the blue, green, red, red-edge, and near-infrared bands acquired by multispectral cameras, respectively.

#### 2.5.2 Binarization method for individual vegetation indices

In this study, the Otsu’s method (Otsu, 1979) was used to segment the VI images to extract cotton seedlings and other features. After completing visual interpretation, the coordinates of the four features were extracted to validate the accuracy by comparing with the *in-situ* measurement results. Then, the VIs with good performance were selected as the key VIs through accuracy validation and visual inspection.

#### 2.5.3 Binarization method for the fusion of multiple vegetation indices

In this study, two modeling strategies, Otsu-intersection (OI) and machine learning (ML), were used to fuse key VIs to increase the prediction accuracy.

In OI, the binarized images of six key VIs were intersected. It uses the differential responses of different VIs to features to eliminate noise and increases the signal-to-noise ratio of binarized images.

Three machine learning methods including SVM (Noble, 2006), RF (Breiman, 2001), and KNN (Cover, 1968) were used for modeling.

#### 2.5.4 Accuracy of different binarization methods

This study used the ratio of correctly labeled pixel samples to total pixel samples (*Accuracy*) to evaluate the accuracy. In the segmentation using the Otsu’ method, all 963 pixel samples collected were used for the validation of the accuracy. Two thirds of the pixel samples of each type of feature were used for modeling with machine learning, and the remaining samples were used for accuracy validation (**Table 3**).

**Table 3:** Pixel sample size of the four features.

Surface feature	Modeling set	Validation set	Total
Cotton seedling	200	102	302
Bare soil	150	74	224
Mulch film	158	79	237
PE drip tape	133	67	200
Total	642	322	963


$$Accuracy=\frac{CS}{TS}\times 100\%$$

where *CS* is the correctly labeled pixel samples, and *TS* is the total pixel samples.

### 2.6 Inversion of emergence rate

#### 2.6.1 Morphological filtering

To reduce noise in the binarized images and the interference of leaf overlap, morphological filtering was used for correction (① Filling isolated interior pixels; ② Removing H-connected pixels; ③ Using diagonal fill to eliminate 8-connectivity of the background) (https://www.mathworks.com/help/images/). Besides, objects with pixels below 2 were deleted.

#### 2.6.2 Mesh segmentation and counting

According to the cotton planting pattern in Xinjiang and image quality, this study divided the cotton field into square grids at an interval of 144 pixels (that is, the width of each mulch film). To reduce the impact of differences in the growth of cotton seedlings (i.e., after segmentation, large seedlings may have 16 pixels, while small seedlings may have 5 pixels), the billable function was used to count the number of cotton seedlings in each grid (https://www.mathworks.com/help/images/). Cotton seedlings were all counted as independent individuals. Therefore, the difference in canopy size could not affect the seedling emergence rate prediction. Based on the seeding rate, the cotton seedling emergence rate in each grid was calculated.


$$Emergence\;rate=\frac{NE}{NS}\times 100\%$$

where *NE* is the number of seedlings identified in a grid, and *NS* is the number of seeds sown in the grid.

#### 2.6.3 Accuracy evaluation

The R^2^, MAE, and RMSE were employed to evaluate the accuracy. The higher the R^2^, the lower the MAE and RMSE, the higher the accuracy.


$${\text{R}}^{2}=\;\frac{\sum_{\text{i}=1}^{\text{n}}{\left({\hat{\text{y}}}_{\text{i}}-\bar{\text{y}}\right)}^{2}}{\sum_{\text{i}=1}^{\text{n}}{\left({\text{y}}_{i}-\bar{\text{y}}\right)}^{2}}$$

$$RMSE=\;\sqrt{\frac{\sum_{\text{i}=1}^{\text{n}}{\left({\hat{\text{y}}}_{\text{i}}-{\text{y}}_{\text{i}}\right)}^{2}}{\text{n}}\;}$$

$$MAE=\;\sqrt{\frac{\sum_{\text{i}=1}^{\text{n}}\;{\hat{\text{y}}}_{\text{i}}-{\text{y}}_{\text{i}}}{\text{n}}\;}$$

where *n* is the number of samples for modeling, ${\hat{y}}_{i}$ is the estimate, $y_{i}$ is the measured value, and $\bar{y}$ is the average of measured values.

## 3 Results

### 3.1 Optical characteristics of the four features

**Figure 4:**
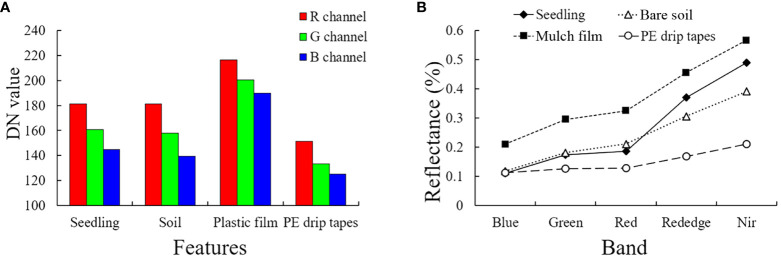
Variations of major features in the RGB **(A)** and multispectral **(B)** images of cotton field.

In the visible images (**Figure 4A**), by visual interpretation, the four features showed the law of R > G > B. The digital number (DN) values of bare soils and cotton seedlings were similar in the red channel, and the DN values of bare soils were slightly lower than those of cotton seedlings in the green and blue channels. The DN values of mulch films were higher than those of cotton seedlings and bare soils due to their high brightness. The PE drip tapes were black in the visible images, so their DN values were lower than those of other features. In the multispectral images (**Figure 4B**), similarly, the reflectance of mulch films was the highest, while that of PE drip tapes was the lowest. The reflectance of bare soils and cotton seedlings were highly similar, especially in the blue and green regions. However, the reflectance of cotton seedlings were lower than that of bare soils in the red region but higher than that of bare soils in the red edge and near-infrared regions. On the whole, there was a significant difference between cotton seedlings and plastic films/PE drip tapes in the images, but there was little difference between cotton seedlings and bare soil.

### 3.2 Segmentation accuracy of each vegetation index

**Figure 5:**
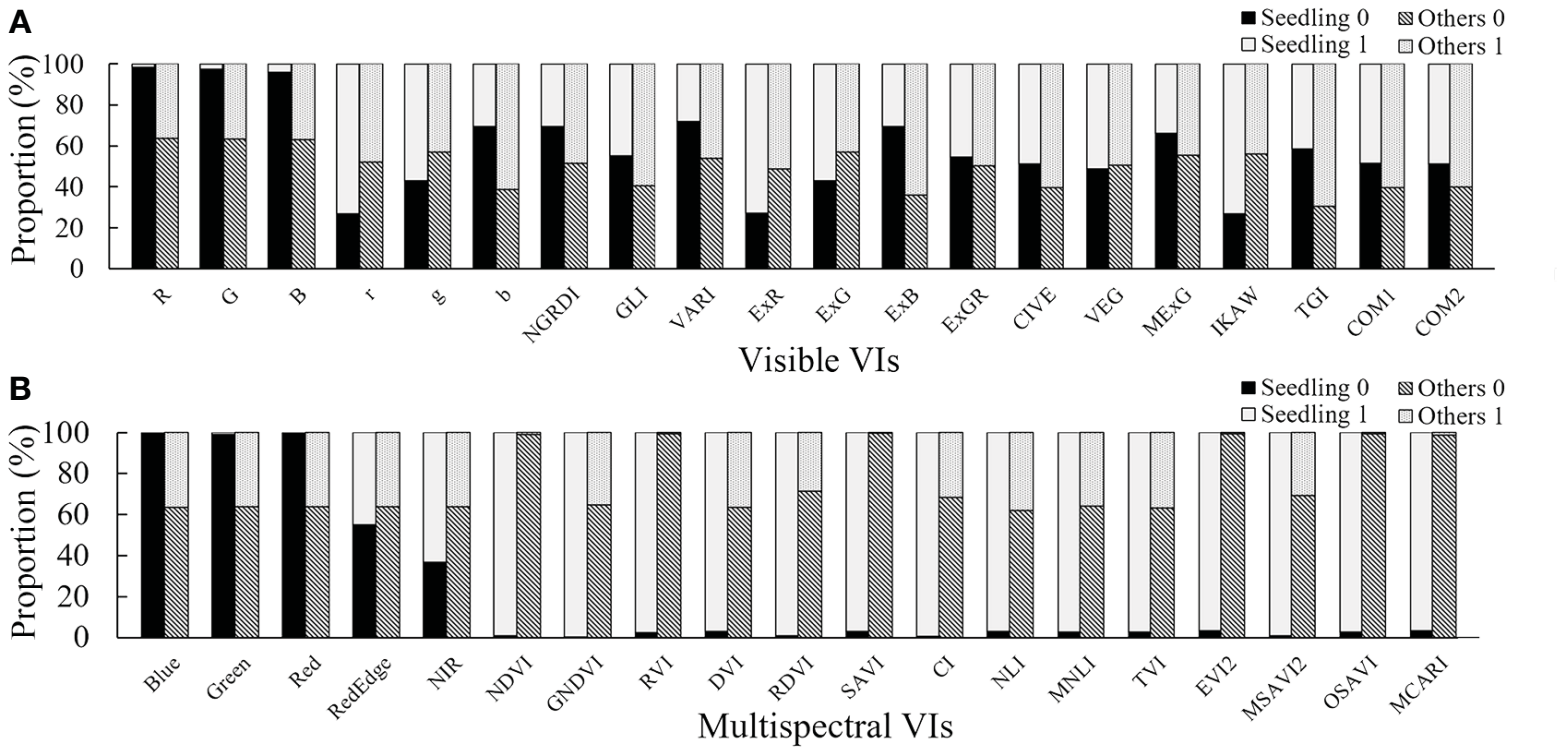
The proportion of cotton seedlings and other features (bare soils, mulch films, and PE drip tapes) in the foreground and background colors in the Otsu’s thresholding results of each vegetation index. Ideally, cotton seedlings have a high proportion in one of 0 or 1, but a very low proportion in the other. Segmentation accuracy of each vegetation index using Otsu’s method. **(A)** RGB vegetation indices; **(B)** Multispectral vegetation indices; Seedling, cotton seedlings; Others, bare soils, mulch films, and PE drip tapes; 0 and 1 are the classification results using Otsu’s method, 0 represents background color, and 1 represents foreground color.

The visible image segmentation results (**Figure 5A**) showed that the visible VIs could not accurately extract cotton seedlings and other features except for the R, G, and B channel data. The R, G, and B channel image segmentation results showed that almost all cotton seedlings were labeled with 0, but there were also a large number of other features that were labeled with 0. So the accuracy using visible images was poor. The multispectral image segmentation results (**Figure 5B**) showed that the segmentation results by Blue, Green, GNDVI, DVI, RDVI, CI, NLI, MNLI, TVI, and MSAVI2 were similar to those of R, G, and B, showing a low accuracy. However, a high accuracy was obtained by using the NDVI, RVI, SAVI, EVI2, OSAVI, and MCARI.

**Figure 6:**
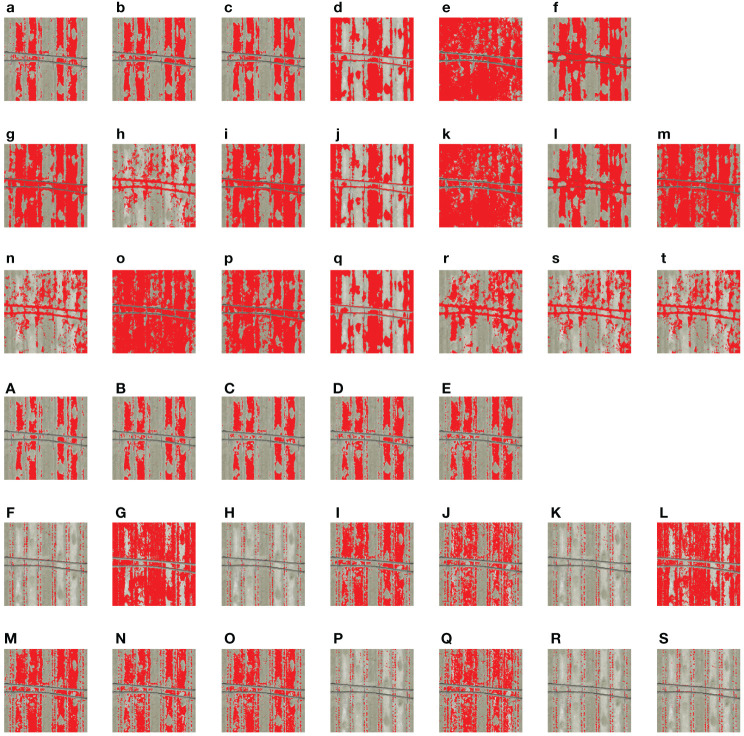
Segmentation results of each vegetation index using Otsu’s method. Red represents the foreground color, and the background color is set to transparent. **(a, B; b, G; c, R; d, b; e, g; f, r; g)** NGRDI; **(h)** GLI; **(i)** VARI; **(j)** ExR; **(k)** ExG; **(l)** ExB; **(m)** ExGR; **(n)** CIVE; **(o)** VEG; **(p)** MExG; **(q)** IKAW; **(r)** TGI; **(s)** COM1; **(t)** COM2; **(A)** Blue; **(B)** Green; **(C)** Red; **(D)** Red-edge; **(E)** NIR; **(F)** NDVI; **(G)** GNDVI; **(H)** RVI; **(I)** DVI; **(J)** RDVI; **(K)** SAVI; **(L)** CI; **(M)** NLI; **(N)** MNLI; **(O)** TVI; **(P)** EVI2; **(Q)** MSAVI2; **(R)** OSAVI; **(S)** MCARI.

The segmentation results for most VIs showed that mulch films caused great interference to the extraction of cotton seedlings (**Figure 6**). After enlarging the six VIs with higher accuracy (NDVI, RVI, SAVI, EVI2, OSAVI, and MCARI), it was found that mulch films and bare soils had more bright points (noise) in the segmentation results of RVI, SAVI, EVI2, and OSAVI. This indicates that these four VIs are more sensitive to soil surface texture. It was also found that PE drip tapes had more bright points (noise) in the segmentation results of NDVI and MCARI. This indicates that these two VIs are easily affected by drip tapes and shadows.

### 3.3 Segmentation accuracy based on the fusion of key vegetation indices

The six key VIs were fused by machine learning (SVM, RF, and KNN) and OI separately, and their segmentation accuracy were compared. It was found (**Table 4**) that the segmentation accuracy of the four models reached more than 96%, and the lowest accuracy was 96.08%. Specifically, the segmentation accuracy of Key VIs-OI and Key VIs-SVM model were 96.69% and 96.08%, respectively, which were slightly lower than that of Key VIs-RF (98.50%) and Key VIs-KNN (98.50%) models. Overall, all four methods had a high segmentation accuracy.

**Table 4:** Segmentation accuracy based on the fusion of key vegetation indices.

Segmentation method	Calibration		Validation	
	Seedling	Others	Seedling	Others
Key VIs-OI	96.69%	99.55%		
Key VIs-SVM	99.50%	98.87%	96.08%	96.36%
Key VIs-RF	98.50%	97.51%	99.02%	98.64%
Key VIs-KNN	98.50%	96.60%	99.02%	98.64%

**Figure 7:**
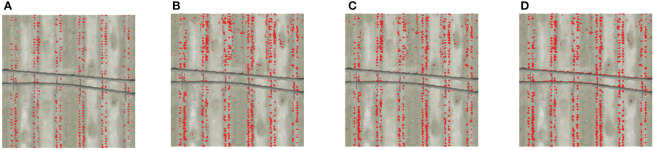
Image segmentation results based on the fusion of key vegetation indices. Red represents the foreground color, and the background color is set to transparent. The four features are divided into two categories, namely cotton seedlings and other features. **(A)** Key VIs-OI; **(B)** Key VIs-SVM; **(C)** Key VIs-RF; **(D)** Key VIs-KNN.

Through the visual interpretation of the image segmentation results (**Figure 7**), it was found that there was more noise for the bare soils and the shadow of drip tapes in the Key VIs-SVM, Key VIs-RF, and Key VIs-KNN segmentation images. Therefore, the segmentation performance of Key VIs-OI model was the optimal.

### 3.4 Segmentation accuracy based on the fusion of all vegetation indices

The data of 1 visible image, 17 visible VIs, 5 multispectral images, and 14 multispectral VIs were used as inputs to construct SVM, RF, and KNN models (**Table 5**). The segmentation accuracy of SVM, RF, and KNN models constructed based on visible VIs was the lowest, and that of visible VIs-RF model was the highest among the visible VI models. The segmentation accuracy of SVM, RF, and KNN models constructed based on multispectral VIs was higher than that of the SVM, RF, and KNN models constructed based on visible VIs, among which the accuracy of multispectral VIs-SVM and multispectral VIs-KNN models were 100% based on the calibration set. The segmentation accuracy of multispectral VIs-RF model was the lowest (98.28%) among multispectral VI models, but it was still much higher than that of visible VI models. After fusing all VIs, it was found that the accuracy was slightly lower than that of multispectral VI models, among which the segmentation accuracy of All VIs-SVM model was the highest (100% based on the calibration set). Overall, the segmentation accuracy of multispectral VI models was the highest.

**Table 5:** Segmentation accuracy based on the fusion of all vegetation indices.

Segmentation method	Number of VIs	Calibration			Validation		
		Seedling	Others	Accuracy	Seedling	Others	Accuracy
Visible Vis							
SVM	20	83.50%	94.56%	91.11%	58.82%	87.73%	78.57%
RF	20	92.00%	93.42%	92.98%	69.61%	86.36%	81.06%
KNN	20	86.50%	89.12%	88.30%	74.51%	82.73%	80.12%
Muti-spectral Vis							
SVM	19	100.00%	100.00%	100.00%	98.04%	99.09%	98.76%
RF	19	99.00%	97.96%	98.28%	98.04%	99.09%	97.83%
KNN	19	100.00%	100.00%	100.00%	99.02%	98.64%	98.76%
All Vis							
SVM	39	100.00%	100.00%	100.00%	97.06%	99.09%	98.45%
RF	39	99.00%	98.64%	98.75%	97.06%	99.55%	98.76%
KNN	39	97.00%	97.73%	97.50%	96.08%	99.55%	98.45%

**Figure 8:**
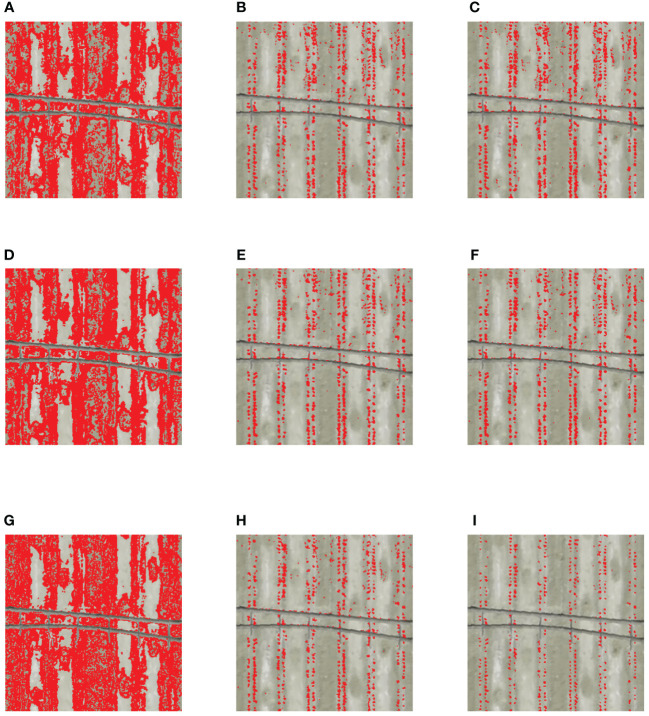
Segmentation results of machine learning. Red represents the foreground color, and the background color is set to transparent. The bare soils, plastic films, and PE drip tapes are set as the background color in the classification, that is, the four features were divided into two categories, namely cotton seedlings and other features. **(A)** Visible VIs-SVM; **(B)** Muti-spectral VIs-SVM; **(C)** All VIs-SVM; **(D)** Visible VIs-RF; **(E)** Muti-spectral VIs-RF; **(F)** All VIs-RF; **(G)** Visible VIs-KNN; **(H)** Muti-spectral VIs-KNN; **(I)** All VIs-KNN.

The segmentation results (**Figure 8**) showed that the visible VI models had a very poor accuracy. The bare soils, the boundary area between bare soils and mulch films, and cotton seedlings were identified as identical features in large quantities, and only PE drip tapes were clearly identified. This indicates that the high reflectance of mulch films increases the upper limit of threshold segmentation and reduces the difference between cotton seedlings and bare soils in the visible region. It is worth noting that when bare soil and plastic film are superimposed, the high reflectance of plastic film increases the brightness of bare soils, making bare soils similar to cotton seedlings in the visible region. The segmentation results of multispectral VI models and the models constructed based on the fusion of all VIs were good, and showed some improvements compared with key VI models. However, there was still some noise. As a whole, the All VIs-KNN model had the optimal segmentation results and the number of noise was small.

### 3.5 Visual inversion of seedling emergence rate in cotton field

**Figure 9:**
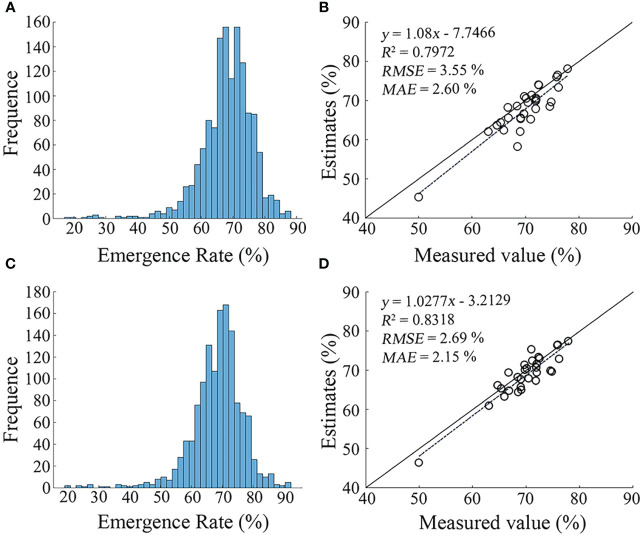
Seedling emergence distribution based on the inversion using the Key VIs-OI and All VIs-KNN models. **(A)** Seedling emergence rate estimated by Key VIs-OI model; **(B)** Validation of the seedling emergence rate inversion accuracy of Key VIs-OI model; **(C)** Seedling emergence rate estimated by All VIs-KNN model; **(D)** Validation of the seedling emergence rate inversion accuracy of All VIs-KNN model.

Based on the results of Section 3.2, 3.3, and 3.4, it was found that the segmentation accuracy of Key VIs-OI and All VIs-KNN models were obviously higher than other models. Thus, these two models were selected. Then, the noise less than 2 pixels were removed by morphological filtering. The counting statistics of cotton seedlings was carried out, to obtain the seedling emergence rate in cotton field based on the sowing density (**Figures 9A, C**). The results showed that the seedling emergence rate was 65% - 75% in most grids, and the mean and median were similar. The mean and median of Key VIs-OI model were 68.12% and 69.21%, respectively, and those of All VIs-KNN model were 68.44% and 69.21%, respectively. Compared with the measured data (**Figures 9B, D**), it was found that the seedling emergence rate estimated by the two models were slightly lower than the measured value. Besides, the accuracy of All VIs-KNN model was higher than that of Key VIs-OI, with a R^2^ of 0.8318, a RMSE of 2.69%, and a MAE of 2.15%.

**Figure 10:**
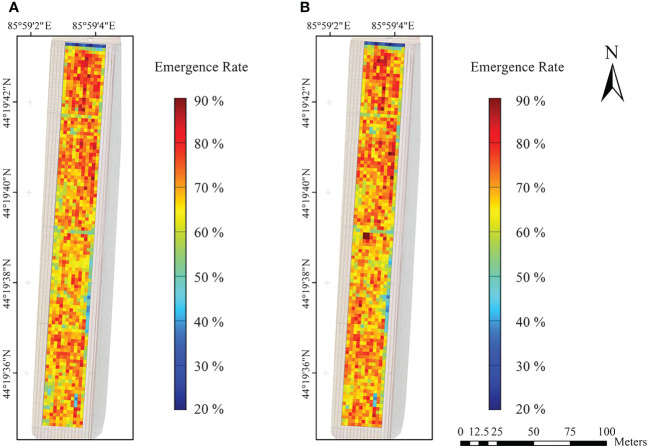
Images of estimated seedling emergence rate in cotton field by machine learning. **(A)** Inversion of seedling emergence rate by the Key VIs-OI model; **(B)** Inversion of seedling emergence rate by the All VIs-KNN model.

The emergence rate estimates obtained by the two models were similar (**Figure 10**). Cotton seed germination around the PE drip tapes was affected by high soil moisture content due to pipe joint water leakage. Cotton seed germination rate was low in the northernmost part of the field bordering the road due to soil hardening. Besides, four low emergence area in the southeast corner of the field was due to insufficient water supply caused by the bending of the drip tapes. On the whole, the spatial distribution of cotton seedling emergence rate in the experimental area was uneven.

## 4 Discussion

This study used cheap consumer-grade drones to obtain images of cotton fields, highlighted the spectral features of cotton seedlings in the images by constructing VIs, and analyzed the response characteristics of four features in the segmentation results of different VIs. After that, a simple and efficient method for cotton seedling emergence rate inversion was proposed, that is, fusing multiple VIs to extract seedlings from the images. This method had a high accuracy. Therefore, it has high application potential in the rapid monitoring of crop seedling emergence rate.

Most previous studies have used a single VI to extract crops from images using the Otsu’s method (Varela et al., 2018; Mhango et al., 2021), but their segmentation results contain a large number of irregularly shaped noise, which is similar to the results of this study. This may be due to that the components in the farmland are very complex, which have some similar strong reflection, diffuse reflection, and other optical properties. Therefore, it is difficult to extract crop seedlings individually during thresholding segmentation (Yeom et al., 2018). In addition, Feng et al. (2020a) reported that soil moisture content and mutual shading of large soil particles might also lead to light and dark changes in soil color, resulting in many noises with a similar shape to crop seedlings, affecting the segmentation accuracy. The widely used ExG was less accurate in this study. This may be due to that film mulching is not adopted in previous studies (Yeom et al., 2018; Banerjee et al., 2021). However, a large number of mulch films on the soil surface showed a higher red/green ratio and blue/green ratio than bare soil, and a higher brightness than crop seedlings during the ExG inversion. This is confirmed by the study results of Dai et al. (2020). Dai et al. (2020) found that the distribution of cotton pixels in different gray scales on ExG images was uniform, which eventually caused the difficulty of crop seedling extraction.

To reduce massive noise in the thresholding segmentation results of VIs, the widely used method is to extract morphological features to match each object in the thresholding segmentation results (Koh et al., 2019). For example, Li et al. (2019) extracted six morphological features as inputs of random forest models for estimating the potato seedling emergence rate, and found that the R^2^ was 0.96. However, the morphological characteristics of seedlings change rapidly with the growth and development of crops. If there is a difference in the time of image acquisition, or if the growth of seedlings becomes faster or slower due to temperature and humidity changes, it will lead to differences between the actual morphological characteristics of seedlings and the model inputs, which ultimately reduces the model accuracy (Zhang et al., 2020). The exploration of spectral differences between cotton seedlings and other features can help overcome these problems. On the one hand, with the growth and development, cotton seedlings’ spectral features change much less than the morphological characteristics. On the other hand, cotton seedlings have obvious spectral differences from other features (Ashapure et al., 2019). The constructed VIs highlighted the characteristics of cotton seedlings and made the difference between cotton seedlings and bare soils/PE drip tapes/mulch films more obvious. Due to VIs have different sensitivity to features (Wei et al., 2019), the fusion of multiple VIs can combine the sensitive information for multiple indices and greatly reduce noise. Similar results were also obtained in the monitoring of crop nutrition status (Xu et al., 2023). Therefore, spectroscopic techniques have great potential in image segmentation and classification.

Varela et al. (2018) and Valente et al. (2020) showed that segmentation of crop seedling images by extracting morphological features and using deep learning relies on high spatial resolution, and low spatial resolution reduced the accuracy of classifiers. Therefore, most scholars make UAVs fly low to obtain high resolution, i.e., Dai et al. (2020); Feng et al. (2020b), and Varela et al. (2018) used 10 m flight altitude to obtain images with a resolution of 0.27 cm, 0.29 cm, and 0.24 cm pixel^-1^, respectively. Wu et al. (2019) used 7 m flight altitude to obtain images with a resolution of 0.06 cm pixel^-1^. However, due to the limited battery capacity of drones, agricultural practitioners have to make trade-offs between size of shooting area and resolution, which limits the application of drones for emergence monitoring (Lin and Guo, 2021). Segmentation of crop seedling images using the spectral differences between cotton seedlings and other features can reduce the dependence of the model on resolution (Wilke et al., 2021). Sankaran et al. (2015) showed that even at 3.0 cm pixel^-1^ resolution, the NDVI value of crops was highly correlated with crop seedling emergence rate (*r* = 0.82). Wilke et al. (2021) compared the images of RGB sensor and multispectral sensor, and found that the effect of spatial resolution on the VI model was lower than that on the model constructed based on visible images in the early stage of leaf development with only two unfolded leaves per plant. When the resolution of RGB images decreased from 0.2 to 0.59 cm pixel^-1^, the MAE decreased from 26 to 55 plants m^-2^; When the resolution of multispectral images decreased from 0.69 cm to 1.38 cm pixel^-1^, the MAE decreased from 24 to 29 plants m^-2^. The *R^2^* obtained in this study was lower than that of Varela et al. (2018); Dai et al. (2020); Feng et al. (2020b), and Wu et al. (2019), but the RMSE (2.69%) and MAE (2.15%) were the lowest. Therefore, the method proposed in this study still has a high accuracy when using images with a low resolution, and can meet the needs of large-scale monitoring and high-precision monitoring at the same time.

The results of this study suggest that the fusion of multiple VIs could obtain purer binary images, which has great potential in crop seedling emergence monitoring. Therefore, in future studies, images with different resolutions will be acquired, to compare the effects of different resolutions on the inversion accuracy of deep learning technology, template matching, and the method used in this study, to obtain an appropriate resolution that can meet the needs of both flight area and spatial resolution in production practice. In addition, the accuracy of the method proposed in this study in monitoring the seedling emergence of other crops needs to be tested in the future.

## 5 Conclusion

This study proposed a method to quickly and intuitively invert cotton seedling emergence rate in cotton field. That is, the remote sensing images of cotton seedlings were acquired by drones to construct the visible VIs and multispectral VIs. Then, multiple VIs were fused to obtain pure binary images. After that, the cotton field was meshed and the cotton seedlings were counted, to obtain the image showing the distribution of cotton seedling emergence rate in the cotton field. The following conclusions were drawn.

• Visible images were susceptible to the influence of high-reflectance features, and the segmentation accuracy based on visible images was lower than that of multispectral images.
• The crop seedling segmentation accuracy based on the fusion of multiple VIs was higher than that of a single VI. Especially, the Key VIs-OI(*R^2^ = *0.7972, *RMSE* = 3.55%, *MAE* = 2.60%) and the All VIs-KNN(*R^2^ = *0.8318, *RMSE* = 2.69%, *MAE* = 2.15%) models had an obviously high segmentation accuracy.
• This study broadened the selection range of VIs and achieved purer crop seedling extraction from images. The proposed method has very low requirements for hardware, and can serve as a low-cost and powerful tool for practitioners to monitor crop seedling emergence. This study will provide technical support for modern agricultural management.

## Data availability statement

The original contributions presented in the study are included in the article/supplementary material. Further inquiries can be directed to the corresponding authors.

## Author contributions

TL: Writing – original draft, Conceptualization, Data curation, Investigation, Methodology, Validation, Visualization. HW: Conceptualization, Funding acquisition, Methodology, Project administration, Supervision, Writing – review & editing. JC: Conceptualization, Investigation, Methodology, Project administration, Supervision, Writing – review & editing. WW: Data curation, Formal analysis, Validation, Writing – review & editing. WL: Formal analysis, Writing – review & editing. MJ: Investigation, Writing – review & editing. XS: Investigation, Writing – review & editing. JS: Investigation, Writing – review & editing. JW: Investigation, Writing – review & editing. XL: Conceptualization, Funding acquisition, Project administration, Resources, Writing – review & editing. LZ: Conceptualization, Funding acquisition, Methodology, Resources, Writing – review & editing.
